# Retraction: MicroRNA-625 inhibits cell invasion and epithelial-mesenchymal transition by targeting SOX4 in laryngeal squamous cell carcinoma

**DOI:** 10.1042/BSR-2018-1882_RET

**Published:** 2023-01-30

**Keywords:** epithelial-mesenchymal transition, laryngeal squamous cell carcinoma, microRNA-625, SOX4

This article is being retracted from *Bioscience Reports* at the request of the Editor-in-Chief and the Editorial Board following receipt of a notification from a reader, alerting the Editorial Board to multiple similarities between the figures in this manuscript and those in other articles. These include similarities between:

- The Figure 4C AMC-HN-8/GAPDH bands with the Fig.6A A549/GAPDH bands from Wang et al. 2019 (doi: 10.1042/BSR20182433) and the Fig.3F GAPDH bands from Chen et al. 2019 (doi: 10.1042/BSR20191050),
- The Figure 2C miR-625 mimics panel with Fig.6E from Xiao et al. 2020 (doi: 10.18632/aging.103762) and Fig.1D from Hu et al. 2019 (doi: 10.1002/2211-5463.12693)
- The Figure 2C mimics control/Tu-177 panel with a panel from Fig.1D from Hu et al. 2019 (doi: 10.1002/2211-5463.12693)
- The Figure 3B mimics control/Tu-177 panel and the 5C miR-625+NC/Tu-177 panel with panels from Fig.4B from Cheng et al. 2019 (doi: 10.1042/BSR20180906)

The authors have been contacted with regards to the retraction and have not responded to the Journal's queries or the concerns raised. Given the extent of the issues raised, the Editorial Board stand by the decision to retract the article.

